# Supplementary material for: Antibiotic resistance, pathotypes, and pathogen-host interactions in Escherichia coli from hospital wastewater in Bulawayo, Zimbabwe
Source: PLoS One. 2023 Mar 2;18(3):e0282273. doi: 10.1371/journal.pone.0282273 (PMC9980749; doi:10.1371/journal.pone.0282273)
Supplement: S1 Table — (DOCX) [file pone.0282273.s001.docx]

**S1 Table. *E. coli* primers and reference strains (positive controls) used in PCR reactions**

| **Pathogen** | **Name of gene** | **Primer sequence (5’- 3’)** | **Reference strain** | **References** |
| --- | --- | --- | --- | --- |
| EPEC/EHEC | *eaeA* | (F) ATGCTTAGTGCTGGTTTAGG | DSM8695 | Stanilova et al. (2011) |
|  |  | (R) GCCTTCATCATTTCGCTTTC |  |  |
| EAEC | *éagg* | (F) AGACTCTGGCGAAAGACTGTATC | DSM10974 | Pass and Odedra (2004) |
|  |  | (R) ATGGCTGTCTAATAGATGAGAAC |  |  |
| EIEC | *ípaH* | (F) GTTCCTTGACCGCCTTTCCGATACCGTC | DSM9025 | Vidal et al. (2005) |
|  |  | (R) GCCGGTCAGCCACCCTCTGAGAGTAC |  |  |
| EHEC | *fliCH7* | (F) TACCATCGCAAAAGCAACTCC | O157H7 | Cebula et al. (1995) |
|  |  | (R) GTCGGCAACGTTAGTGATACC |  |  |
|  | *sxt* | (F) GAGCGAAATAATTTATATGTG |  |  |
|  |  | (R) TGATGATGGCAATTCAGTAT |  |  |
| ETEC | *St* | (F) TTTCCCCTCTTTTAGTCAGTCAACTG | DSM10973 | Stacy-Phipps et al. (1995) |
|  |  | (R) GGCAGGATTACAACAAAGTTCACA |  |  |
|  | *Lt* | (F) TGCTATGTGCATACGGAGC |  |  |
|  |  | (R) CCATACTGATTGCCGCAAT |  |  |

**Key**: F – Forward primer; R – Reverse primer (adapted from Mbanga et al., 2020, <https://doi.org/10.1186/s12866-020-02036-7>)
