# Supplementary material for: Antibiotic resistance, pathotypes, and pathogen-host interactions in Escherichia coli from hospital wastewater in Bulawayo, Zimbabwe
Source: PLoS One. 2023 Mar 2;18(3):e0282273. doi: 10.1371/journal.pone.0282273 (PMC9980749; doi:10.1371/journal.pone.0282273)
Supplement: S1 Fig — Sample collection was done from site F, the intersection point of A (Children’s ward sewer), B (Intensive Care Unit sewer), C (Burns ward sewer), D (Casualty sewer), and E (Female ward sewer). (DOCX) [file pone.0282273.s002.docx]

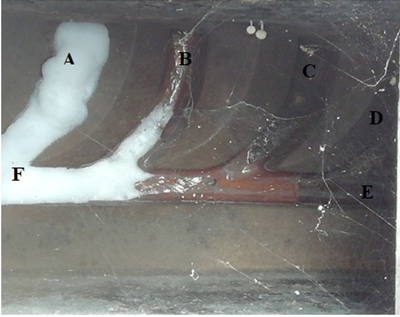


**S1 Fig.** Samples were collected from site F, the intersection point of A (Children’s ward sewer), B (Intensive Care Unit sewer), C (Burns ward sewer), D (Casualty sewer), and E (Female ward sewer)
