# Supplementary material for: Antibiotic resistance, pathotypes, and pathogen-host interactions in Escherichia coli from hospital wastewater in Bulawayo, Zimbabwe
Source: PLoS One. 2023 Mar 2;18(3):e0282273. doi: 10.1371/journal.pone.0282273 (PMC9980749; doi:10.1371/journal.pone.0282273)
Supplement: S2 Fig — (DOCX) [file pone.0282273.s003.docx]

M NC PC 1 2 3 4 5 6 7 8 9 10 11 12 13 14 15 16


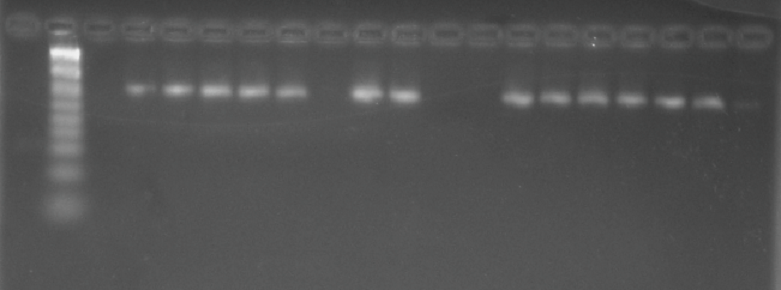


700bp

**S2 Fig.** Representative amplicons obtained by PCR for isolates tested for the *lt* gene, with the expected size of 708bp. Lane M: MWM (Invitrogen 1kb plus ladder), Lane NC: Negative control, Lane PC: Positive control (DSM10973), Lane 1-4; 6-7; 10-16: isolates that were positive for the *lt* gene and Lanes 5, 8 and 9: Samples that are negative for the *lt* gene.
