# Supplementary material for: Antibiotic resistance, pathotypes, and pathogen-host interactions in Escherichia coli from hospital wastewater in Bulawayo, Zimbabwe
Source: PLoS One. 2023 Mar 2;18(3):e0282273. doi: 10.1371/journal.pone.0282273 (PMC9980749; doi:10.1371/journal.pone.0282273)
Supplement: S3 Fig — (DOCX) [file pone.0282273.s004.docx]

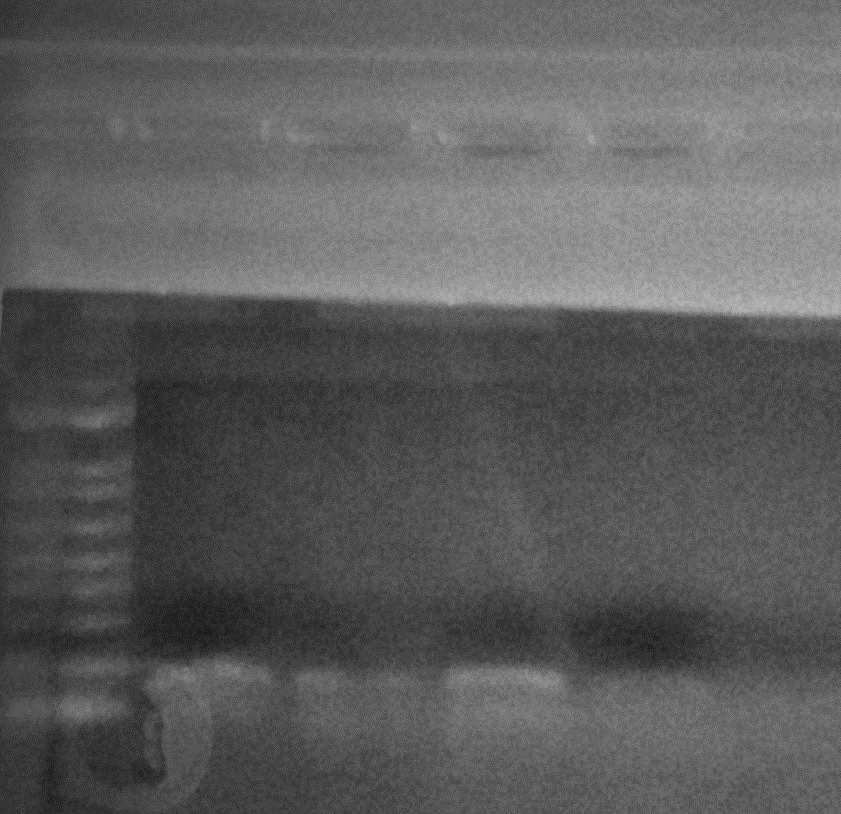


200bp

M PC 1 2 3 NC

**S3 Fig.** Amplicons obtained by PCR for isolates tested for *eagg* gene with the expected size of 194bp, Lane M: MWM Lane PC: positive control (DSM10974), Lane N: Negative control, Lane 1 and 2 isolates that were positive for the *eagg* gene; Lane 3 a negative isolate; NC negative control
