# Supplementary material for: Antibiotic resistance, pathotypes, and pathogen-host interactions in Escherichia coli from hospital wastewater in Bulawayo, Zimbabwe
Source: PLoS One. 2023 Mar 2;18(3):e0282273. doi: 10.1371/journal.pone.0282273 (PMC9980749; doi:10.1371/journal.pone.0282273)
Supplement: S4 Fig — (DOCX) [file pone.0282273.s005.docx]

M PC1 PC2 1 2 NC


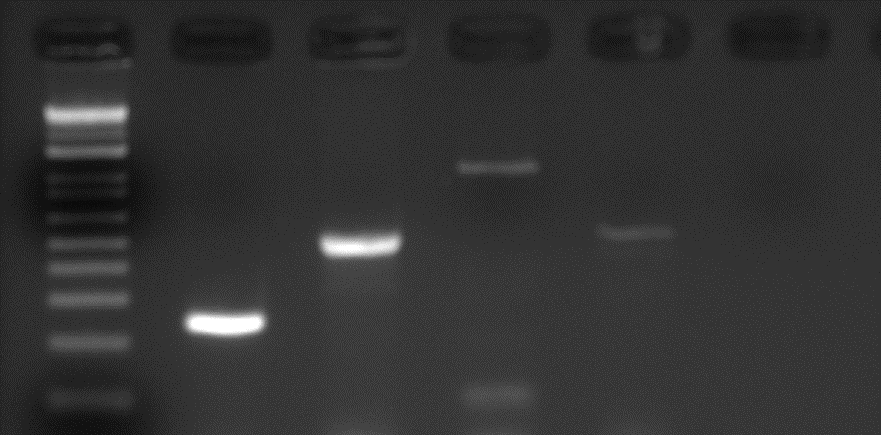


200bp

300bp

500bp

**S4 Fig.** Amplicons obtained by PCR for isolates tested for the *eaeA* and *stx* gene, with the expected size of 248bp and 478bp. Lane M: Molecular ladder (1kb), Lane P1: *eaeA* positive control (DSM8695), Lane P2: Positive control for *stx* (0157:H7), Lane 1: sample negative for *eaeA*, Lane 2: isolate positive for the *stx* gene, and Lane NC: Negative control.
