# Supplementary material for: Antibiotic resistance, pathotypes, and pathogen-host interactions in Escherichia coli from hospital wastewater in Bulawayo, Zimbabwe
Source: PLoS One. 2023 Mar 2;18(3):e0282273. doi: 10.1371/journal.pone.0282273 (PMC9980749; doi:10.1371/journal.pone.0282273)
Supplement: S5 Fig — (DOCX) [file pone.0282273.s006.docx]

M PC NC 1


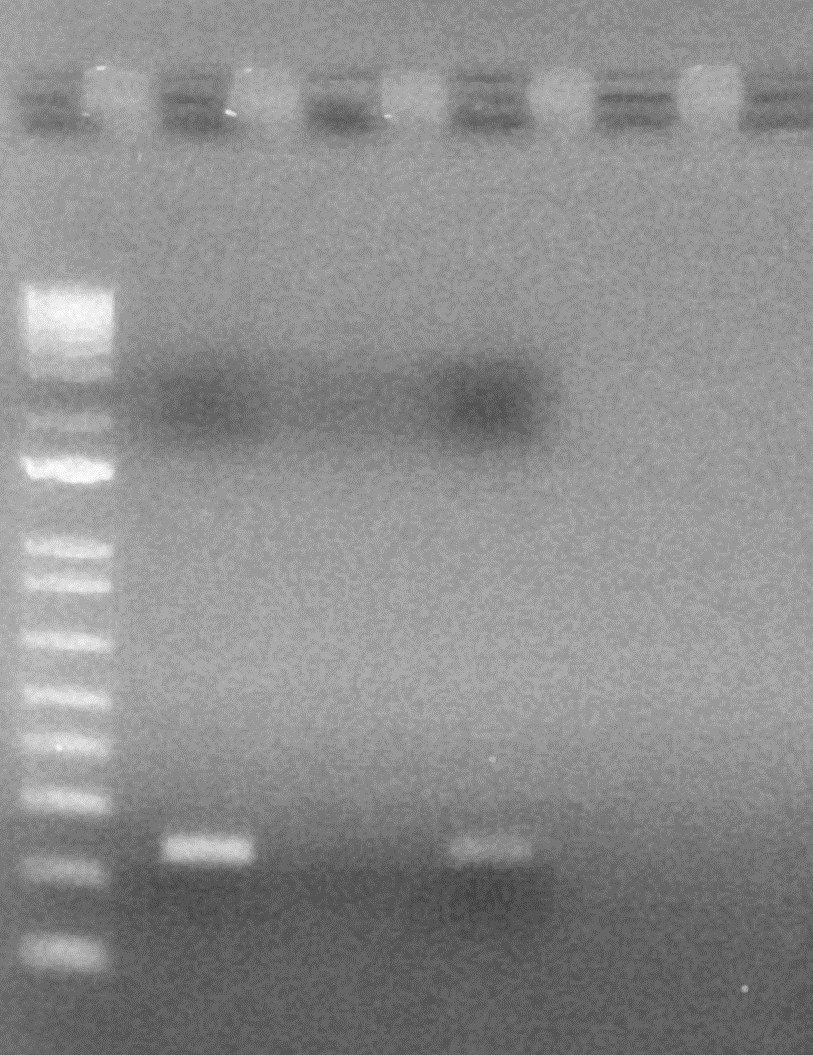


200bp

300bp

**S5 Fig.** Amplicons obtained by PCR for isolates tested for the *eaeA* with the expected size of 248bp, Lane M: MWM (1kb ladder), Lane NC: Negative control, Lane PC: positive control (DSM8695), Lane 1: isolate positive for the *eaeA* gene.
