# Supplementary material for: Antibiotic resistance, pathotypes, and pathogen-host interactions in Escherichia coli from hospital wastewater in Bulawayo, Zimbabwe
Source: PLoS One. 2023 Mar 2;18(3):e0282273. doi: 10.1371/journal.pone.0282273 (PMC9980749; doi:10.1371/journal.pone.0282273)
Supplement: Attachment — Submitted filename: PLOS ONE REBUTTAL.docx [file pone.0282273.s007.docx]

Dear Dr. Rouby

Academic Editor: PLOS ONE

**RE:** Rebuttal letter

Title: Antibiotic resistance, pathotypes, and pathogen-host interactions in Escherichia coli from hospital wastewater in Bulawayo, Zimbabwe. PONE-D-22-26831.

Please find below a Table in response to the comments of both reviewers. The changes to the manuscript are indicated in the text by tracked changes (reviewer 1) and in tracked changes and yellow highlight (reviewer 2).

| **Reviewer’s comments** | **Response/Rebuttal** |
| --- | --- |
| **Editor’s comments** | |
| Please ensure that your manuscript meets PLOS ONE's style requirements, including those for file naming | We have made the necessary amendments to meet PLOS ONE style requirements |
| We note that you have provided funding information that is not currently declared in your Funding Statement. However, funding information should not appear in the Acknowledgments section or other areas of your manuscript. We will only publish funding information present in the Funding Statement section of the online submission form.  Please remove any funding-related text from the manuscript and let us know how you would like to update your Funding Statement. Currently, your Funding Statement reads as follows:  "The author(s) received no specific funding for this work."  Please include your amended statements within your cover letter; we will change the online submission form on your behalf. | The funding information has been removed from the Acknowledgement section. We have included the amended funding statement in the cover letter. |
| Your ethics statement should only appear in the Methods section of your manuscript. If your ethics statement is written in any section besides the Methods, please move it to the Methods section and delete it from any other section. Please ensure that your ethics statement is included in your manuscript, as the ethics statement entered into the online submission form will not be published alongside your manuscript. | The Ethics statement has been moved to the Methods and materials section. |
| Please include your tables as part of your main manuscript and remove the individual files. Please note that supplementary tables (should remain/ be uploaded) as separate "supporting information" files. | The tables have been included in the main manuscript. |
| **Reviewer 1** | |
| Some parts of the text need grammatical and topographical corrections. Please do it using a native English speaker. | We used a language editor Grammarly to edit the manuscript & sought additional assistance to improve the grammar |
| How many samples were collected? | Four wastewater samples were collected (duplicate samples per week for a month). This is mentioned in line 108. |
| In the method section, there was no information about the type of collected samples (liquid or solid wastewater), method of sampling, number of samples, and other information | The type of collected sample is mentioned. line 105 ” The sampled sewerage mains receives liquid waste…”  Method of sampling has been added – line 108  Number of samples – line 108 |
| The authors utilized only the Gram staining and oxidase test to approve *E. coli* isolates. It is not enough to carefully confirm the *E. coli* isolates | The selective media Eosin Methylene blue was used for isolation, and confirmation was primarily based on the PCR. More biochemical tests could have been necessary had we not done molecular confirmation. |
| Why did the authors use uidA (β-D glucuronidase) gene for E. coli identification? Why they didn’t use 16SrRNA? | The *uidA* gene sequence is unique to E. coli (Bej et al., 1991, Appl Environ Microbiol 57:1013–1017). This means it can be routinely used for the identification of *E. coli* species {Green et al. 1991, <https://doi.org/10.1016/0167-7012(91)90046-S> } {Maheux et al. 2009, Water Res 43:3019–3028}, {Janezic et al. 2013, Open Microbiol. J. 7:9–19.}, { Naganandhini et al. 2015, 10.1371/journal.pone.0130038}, {Mbanga et al., 2021, 10.1089/mdr.2020.0380}  - I6SrRNA works for molecular identification if it is married with PCR amplicon sequencing. |
| Please completely determined the method used for DNA extraction. | Heat lysis protocol is the boiling method a reference has been added. Line 133. |
| How the quality and quantity of extracted DNA samples were determined? | The quality and quantity of DNA were determined using gel electrophoresis and a molecular weight marker. This has been included in the manuscript. Lines 133 – 139. |
| Are you sure that E. coli ATCC® 25922 is positive for the uidA gene? | Yes. Very positive. As previously reported in many papers. (Titilawo et al., 2015, <https://doi.org/10.1186/s12866-015-0540-3>), { Naganandhini et al. 2015, 10.1371/journal.pone.0130038}, {Mbanga et al., 2021, 10.1089/mdr.2020.0380}, |
| What about the negative control in the first PCR? | The negative control is mentioned in line 145 |
| Please add positive and negative controls for the pathotyping of E. coli strains. | The positive controls are referred to as reference strains in line 152. To ensure there is no ambiguity we have added positive controls to the sentence in lines 152 – 153.  The negative control is mentioned in line 160. |
| Please add the findings of disk diffusion for your applied control E. coli ATCC 25922. | We feel it is unnecessary to include this result in the main manuscript as the results of the disc diffusion assay for this control strain are well known. The isolate is susceptible to most antibiotics. Should the reviewer insist on seeing these results, they can be availed via email. |
| In my opinion, both supplementary files (Table and figure) should add to the text of the paper not as supplementary | As suggested, we have included supplementary table S2 in the main text as Table 1. We, however, do not see the value of including supplementary Table S1 in the main text as the Table was previously published as supplementary material (Mbanga et al., 2020, <https://doi.org/10.1186/s12866-020-02036-7> ). S1 Fig. is a picture of the wastewater collection site (drainage) in the main sewer of the hospital and is hardly referred to in the main text. Hence, we believe it is appropriately placed in the supplementary materials section. |
| -In the method used for pathotyping, the pathotypes assayed for included EHEC, EPEC, EIEC, EAEC, and ETEC were identified only by PCR. However, it was not enough. Because the authors determined the EHEC/EPEC strains as those that contained eaeA gene (and stx and fliCH7 for the EHEC strains (However, how do they differentiate from EHEC and EPEC?)), EAEC as those contained the eagg gene, EIEC gene as those contained ipaH gene, ETEC those contained st and lt genes (it is not determined that both of them or only one of them?). In my opinion, the method should do with more accurate details. It is also important to add controls. | The method used to pathotype *E. coli* in our study is a standard protocol that has been reported in several studies. We list a few below.   - <https://doi.org/10.1016/j.scitotenv.2016.05.155>. - <https://doi.org/10.1111/1469-0691.12646>. - <https://doi.org/10.1186/s13099-019-0290-0>. - Omar and Barnard, 2014, World J Microbiol Biotechnol. 2014;30(10):2663–71 - Adesifoye et al. 2016, Microbiology open. 2016;5(1):143–51   We stand guided on what additional assays the reviewer feels should have been done. |
| Please add the PCR figure of genes. | These have been added as supplementary material. |
| Which bacterial concentration was used in the disk diffusion? | The antibiotic susceptibility testing and interpretation of results were done according to CLSI guidelines. This has been clarified in lines 182 -183. Bacterial concentration should be equivalent to a 0.5 MacFarland standard. |
| Please determine the antibiotic resistance pattern based on the pathotype of E. coli strains, too. | The manuscript includes antibiotic resistance patterns based on the *E. coli* pathotype in Table 4. |
| As Chloramphenicol is a forbidden antibiotic. Why 68.1% of isolates harbored resistance against it? | We need clarification on the question. Is the expectation that there should be no resistance to chloramphenicol because it is banned? Resistance mechanisms are determined by a myriad of factors, including mobile genetic elements which carry a variety of resistant determinants that work against a plethora of antibiotics. Antibiotic resistance is influenced by many factors we might not fully understand for instance, why we find antibiotic-resistant bacteria in pristine environments. |
| Please add more details about the exact meaning of week 1, week 2 , … | This request is not clear. We sampled for four weeks, from week 1 to week 4. |
| Please add the study limitation | Limitations are mentioned in lines 451 – 456. |
| Please add more interpretations about the findings of the study in the discussion section. | The discussion has been expanded as suggested. |
| **Reviewer 2** | |
| Limitation in sample size…, why is only one of each pathotype selected for the adherence and invasion assays? I suggest it to be done in triplicate where possible, that will add more validation to support the conclusions | Thank you for your comment. Indeed using more isolates would have improved the validity of the results. However, we used one isolate from each pathotype mainly because we had only one EHEC isolate. We reasoned that our experimental isolates and the positive controls used in the study would give us reliable results. |
| Results are very brief, mostly just a few sentences followed by a Table or Figure. I suggest the authors to combine the results and discussion, especially that they still reference figures in the discussion section. | We have added two tables to the manuscript and expanded the results section. We hope this is sufficient for the manuscript to retain the individual results and discussion sections. |
| The authors included the statistical analysis in the methods, but interrogation is lacking in the text, statistically significant results must be clearly highlighted within text and in the figures/tables | This has been done in the figures (Fig. 3a-c) and intext. |
| Abstract  Statement of public health concern should be removed from the last line and revised to fit as first sentence in the abstract, followed by the aims of the study. | The statement has been removed from the abstract entirely. |
| Introduction  Line 63-64: remove “acting as a hotspot for” and replace with “facilitating”. Use ARGs acronym as already defined in line 58 | The suggestions have been implemented, facilitating added - line 67. ARG acronym used – line 67 |
| Line 77: Predatory bacteria; is defined as bacteria that eat other bacteria, I do not think it was used here in that context. Consider a most suitable term | The term predatory has been replaced by pathogenic – line 83 |
| Sample collection:  Not clear what the duplicate samples were collected for, were they all analyzed independently? | The duplicate samples were pooled and analysed as one composite sample. Lines 110 – 111. |
| Line 95: Supplementary Figure S1, not sure what is being described in the figure? Is this the original photo, maybe a diagram will depict better  The figure shows different sites, A-F, the results will be more interesting if the samples were collected from each site? | Figure S1 is a photograph of the main sewer where wastewater was collected. It shows that effluent from different wards merges into one at the sampled point. We concur that the wastewater could have been sampled per respective ward, however, this was out of the scope of this study but should be considered in future studies. |
| Authors mention that the effluent sample was serially diluted from -1 to -10, 100uL was spread-plated for only -1 to -5, why was -6 up to -10 was not plated or the point of dilution upto -10 | Thank you for this observation. We made dilutions up to -5 not -10 the mistake has been corrected. – Line 121. |
| Line 114: Is there a reference for the standard heat lysis protocol? If not briefly describe the protocol.  adherence and invasion assay | A reference has been added – line 133 |
| line 153-154: specify the strains of positive controls used, were they ATCC or obtained elsewhere. Are these the ones mentioned as clinical strains in Figure 3, 4, and 5? | A statement specifying the positive controls/reference strains has been added. DSM isolates were used. Lines 152 -154. The same reference isolates were used for the infectivity study. |
| Figures 3-5 should also be combined into 1 figure, Figure 3 to be 3A, figure 4 to be 3B, and figure 5 to be figure 3C, and all graphs should use the same scale (e.g. 0.0 logcfu/mL to 10 logcfu/mL for adherence, invasion, and 0.0 to 5.0 logcfy/mL for intracellular survival ) for better comparison among the different pathotypes in three different assays | Because of the differences in the results of the assayed pathotypes the software gave us the best output by determining the scale for the Y-axis. We have, however, reorganised the Figs to 3a. showing the adherence assay, 3b. showing the invasion assay and 3c. showing the intracellular survival assay for easy comparison among the different pathotypes in the three different assays. Fig.3a-c. |
| Why is ETEC (BHE16) not tested for adherence? | The isolate was tested for adherence but yielded negative results. Lines 302-303 |
| Why is EAEC(BHE95) not tested for intracellular survival? | The isolate was tested for adherence but yielded negative results. Lines 306-307 |
| Results and discussion Line 197, 252, 257 : incorporate n=…, to clearly indicate the number of isolates | This has been amended lines 263 – 265. |
| Line 264, 298 name of the genes and strains should be in italics | This has been amended in the entire document. |
